# Large Language Models as a New Tool for Therapists in Internet-Based Cognitive Behavioral Therapy: Blinded Clinician Rating Pilot Experiment

**DOI:** 10.2196/96835

**Published:** 2026-08-26

**Authors:** Thomas Tandrup Lamm, Arthur Bran Herbener, Oliver Rønn Christensen, Malene Flensborg Damholdt, Kaare Bro Wellnitz, Heidi Frølund Pedersen, Lisbeth Frostholm

**Affiliations:** 1 Department of Clinical Medicine Aarhus University Aarhus N Denmark; 2 Department of Functional Disorders Aarhus University Hospital Aarhus N Denmark; 3 Department of Psychology and Behavioral Sciences Aarhus University Aarhus C Denmark

**Keywords:** artificial intelligence, digital mental health, eHealth, functional somatic disorder, generative AI, human-AI comparison, iCBT, internet-based cognitive behavioral therapy, large language models, psychotherapy, telemedicine, text-based therapy

## Abstract

**Background:**

Internet-based cognitive behavioral therapy (iCBT) is an effective and scalable alternative to face-to-face psychotherapy, but its reach is constrained by the time therapists spend reviewing patient input and manually drafting written responses. Studies suggest that large language models (LLMs) may be capable of generating high-quality therapeutic text, with the potential to support therapists in delivering treatment. Their suitability as therapist-support tools in structured iCBT, however, remains insufficiently studied.

**Objective:**

This study aims to assess the quality of LLM-generated iCBT responses to patient messages by comparing them to the quality of responses produced by humans.

**Methods:**

In a preregistered blinded clinician rating experiment, experienced clinicians assessed the quality of human-produced vs LLM-generated therapist responses within a simulated iCBT treatment for functional somatic disorder. Raters were exposed to a stimulus material consisting of 5 fictitious patient messages, each paired with 1 human and 1 LLM-generated response. Raters assessed message/response pairs on 5 quality dimensions (overall quality, helpfulness, empathy, professionalism, and protocol adherence) and were asked to indicate the source of the response (human/LLM). Analyses were primarily descriptive, supplemented by exploratory statistical tests and descriptive thematic content analysis of open-ended text fields. The full preregistered study protocol is available at Open Science Framework.

**Results:**

A total of 61 raters provided data, while 54 were eligible and included for analysis. Human- and LLM-generated responses were rated similarly across quality dimensions on a 1-5 scale: overall quality (LLM: mean 4.00, SD 0.54 vs human: mean 3.96, SD 0.53; *d*=0.06), helpfulness (LLM: mean 3.85, SD 0.57 vs human: mean 3.93, SD 0.49; *d*=0.13), professionalism (LLM: mean 4.25, SD 0.53 vs human: mean 4.11, SD 0.53; *d*=0.24), protocol adherence (LLM: mean 4.13, SD 0.52 vs human: mean 4.13, SD 0.54; *d*=0.03) and empathy (LLM: mean 4.31, SD 0.47 vs human: mean 4.08, SD 0.50; *d*=0.42). Raters correctly identified the source of human-generated responses (mean 79%, SD 19.65%) more accurately than LLM-generated responses (mean 63%, SD 21.30%). In all, 30/54 (55%) raters responded to one or more open text fields. Qualitative analysis indicated that LLM-generated responses were perceived as polished but also generic and at times excessively empathetic.

**Conclusions:**

LLM-generated responses were judged to be of comparable quality to those written by human therapists, though qualitative feedback indicated they were at times generic and insufficiently challenging. These findings provide initial support for the feasibility of using LLMs as therapist-support tools in iCBT, but further research is needed to determine whether their integration yields tangible clinical and organizational benefits.

## Introduction

Mental health conditions represent a globally rising public health challenge [1]. Developing scalable and effective interventions to address central barriers to treatment within mental health service delivery remains paramount [1-3]. The 21st century has seen a gradually accelerating rate of digital innovation within mental health care delivery [2]. Among these, internet-based cognitive behavioral therapy (iCBT) has emerged as a new therapeutic modality. Here, patients access information and therapeutic exercises relevant to their mental health condition online while a therapist supports them via telephone or video consultations and text messages [4]. iCBT has been shown to be a more accessible treatment alternative to face-to-face treatment [5], resulting in large-scale implementations in several countries [6].

The emergence of large language models (LLMs) has opened a potentially new chapter in the delivery of digital mental health services where every individual could have 24/7 access to their own personalized digital therapist at a fraction of the cost of face-to-face psychotherapy [7-11]. Several experimental studies comparing human and LLM-generated responses in text-based counseling and advisory contexts find that LLM responses are rated as comparable to human responses on overall quality and helpfulness, and are in certain cases rated better than human responses [12-15]. LLMs especially tend to generate text responses that convey more empathy or compassion than human responses [14,16,17]. Certain studies further indicate that raters struggle to reliably distinguish LLM-generated responses from human-written ones [12,16].

Evidence from clinical testing of the therapeutic capacities of LLMs, however, remains limited [18]. The application of LLMs in mental health contexts further raises substantial safety concerns [10,19,20]. Individuals seeking help may be in a vulnerable state and thus particularly sensitive to suggestions from therapists. Yet, LLMs are inherently probabilistic and are known to hallucinate and have sycophantic tendencies, potentially reinforcing users’ beliefs and preferences [21]. Consequently, LLMs may at times generate inappropriate responses, reinforce avoidance behavior, validate psychotic beliefs, or encourage suicidal ideation [22]. In addition, LLMs may reproduce biases present in training data, perpetuating stigma and disparities in mental health care [10]. These risks raise important ethical and legal concerns and complicate the implementation of direct patient-LLM interaction in clinical settings. In a European context, for example, LLM-based health interventions are classified as high-risk applications under the European Union Artificial Intelligence Act, thereby requiring human oversight [23].

To facilitate integration of LLMs into clinical contexts, a gradual stepwise implementation strategy has been suggested [7,8,11,24]. LLMs may initially be deployed for narrowly defined tasks within highly controlled settings supporting human therapists. This would allow for the gradual accumulation of systematic evidence on the effectiveness and risk profile of LLM technologies, enabling responsible implementation and scaling while minimizing undue risks to patients.

iCBT as a treatment format is particularly well suited for the implementation of LLM technologies. The therapist workflow in iCBT is almost entirely digital, and manually drafting text responses to patients represents a major portion of the workload [4,25]. Given previous studies indicating that LLMs are capable of generating empathic and helpful responses within a therapeutic context, LLMs may be able to assist therapists by generating responses that human therapists could then review and edit before sending [26,27]. This could drastically reduce clinicians’ time per patient, thus enabling a reallocation of resources to patients that may need more intense assistance. It could also allow the provision of much closer support, potentially facilitating stronger patient engagement in iCBT without having to devote additional therapeutic resources [28,29]. LLMs could also help improve the quality of iCBT responses by helping therapists consider alternative intervention strategies or by providing suggestions for how therapists can convey more empathy for patients [30,31]. Framed in this way, the value of LLM-based therapist support does not lie in automating care, but in enhancing and extending professional capacity.

Realizing this potential would, however, require several conditions to be met: LLM-generated responses must be clinically useful and comparable in quality to those written by human therapists, and the technology must provide tangible workflow benefits such as reducing the time required to draft responses or improving clinical outcomes. Further, it must be perceived as useful, relevant, and acceptable by both therapists and patients.

Existing work has primarily focused on whether LLMs can generate clinically meaningful responses. However, these studies have largely been conducted in generic or simulated settings, including responses to general life problems [14,17,32], mental health-related questions in advice-column formats [12,16], or textbook examples of therapeutic interactions [13]. In addition, several studies rely on convenience-sampled raters from online platforms (eg, Prolific) to evaluate response quality rather than clinically trained mental health professionals. Thus, while prior work has examined the extent to which LLMs can provide relevant therapeutic support, their application within a clinical iCBT context has not yet been investigated.

The aim of this study is to assess the quality of LLM-generated responses to patient messages in an iCBT context by comparing them to the quality of human-produced responses.

To explore the research question, we formed the following exploratory preregistered hypotheses based on the existing literature.

- H1: LLM-generated responses will be rated higher in overall quality than human-generated responses.
- H2: LLM-generated responses will be rated as more empathetic than human-generated responses.
- H3: Raters will not have an above chance ability to correctly identify if a response was generated by a human or an LLM.

## Methods

### Study Design

**Figure 1:**
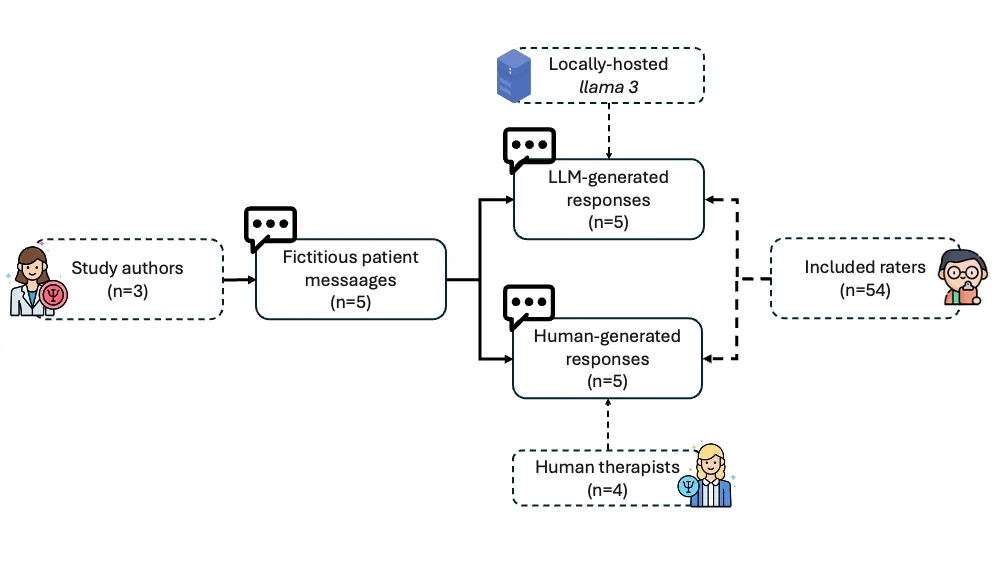
Graphic illustration of research design, including the generation of the stimulus material. LLM: large language model.

To examine whether LLMs can be meaningfully integrated into iCBT workflows for drafting therapist responses to patient messages, a blinded clinician rating experiment was conducted [24]. The experiment consisted of recruiting experienced clinicians to rate pairs of fictitious patient messages and therapist responses on selected quality dimensions. Each message/response pair was designed to emulate treatment interactions within iCBT for functional somatic disorder (FSD), a condition characterized by persistent and impairing somatic symptoms that are not better explained by other psychiatric or somatic diagnoses [33]. One half of the responses were generated by a relevantly prompted LLM and the other half of the responses were written by an experienced human therapist (further detailed in the “Materials” section). Raters were blind to who formulated the responses (human/LLM), allowing for comparison between the LLM and human therapist responses. For an illustration of the design, see Figure 1.

The study was preregistered on May 20, 2025, at Open Science Framework (OSF) [34], where the full study protocol is accessible. Minor adjustments to the preregistration were made regarding (1) the number of patient-therapist correspondences included in the experiment and (2) the wording of one question relating to protocol adherence. The recruitment window was also postponed. All changes were made prior to data collection and are documented in the OSF preregistration.

### Included Raters

To ensure sufficient competence and professional diversity among the included raters, the following eligibility criteria were used: (1) raters must have experience working clinically with patients with FSD or experience in delivering iCBT, and (2) raters must be a clinically active psychologist or medical doctor. The only exclusion criterion was that members of the author group were exempt from participating as raters.

Given the limited size of the target population, it was assumed that achieving a sample large enough for inferential statistical testing would not be feasible. Instead, a time-bounded convenience sampling strategy was used, recruiting as many eligible raters as possible within a predefined recruitment window (September 25, 2025, to November 25, 2025). After this period ended, no additional raters were enrolled, and the analyses were conducted on all eligible data collected.

Recruitment was primarily network-based, targeting university and hospital settings known to employ clinicians and researchers with experience in either FSD or iCBT. Raters were recruited internationally, with a primary focus on European countries.

### Materials

#### Overview

**Figure 2:**
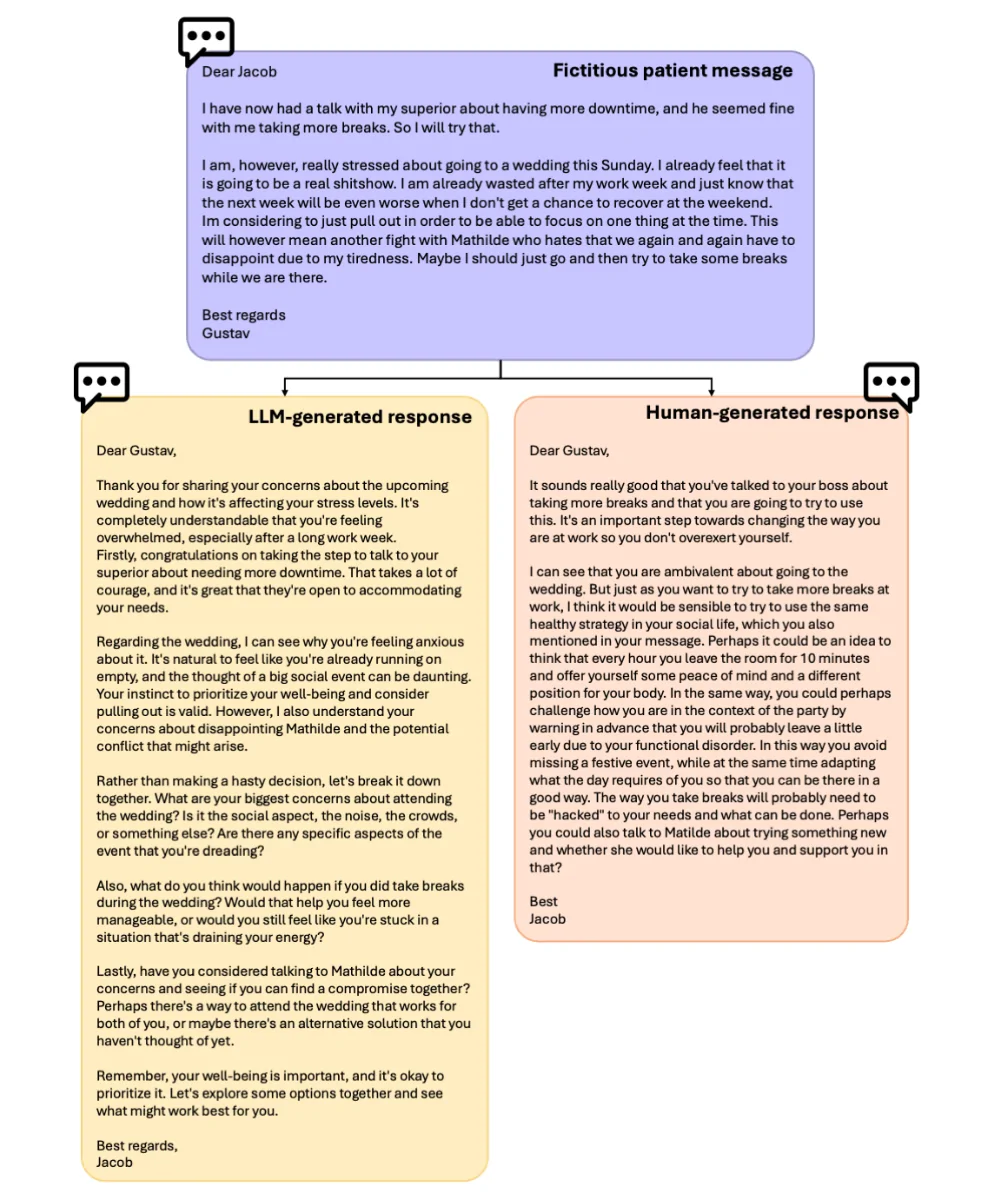
Example of fictitious patient message with a corresponding large language model (LLM)– and human-generated therapist response.

A central feature of the experiment was the stimulus material that the raters were exposed to and rated. The stimulus material consisted of 5 fictitious patient messages, each with an LLM-generated response and a human-generated response (for an example, see Figure 2). The method used to generate each component of the stimulus material is described below.

As LLMs exhibit constrained problem-solving capabilities in low-resource languages (eg, Danish) [35-37], the experiment was conducted in English. The researchers and clinicians who were available for generating therapist responses were, however, all Danish and only had clinical experience working with Danish patients. As such, the fictitious patient messages and the human-generated responses were all initially generated in Danish and then translated into English before the messages and responses were included in the final stimulus material.

Translation was conducted by a qualified translator, double-checked with the authors of the messages, and checked by a native English speaker. During translation, the peculiarities (eg, errors, idioms, and idiosyncrasies) of the original text were retained as much as possible. A graphical illustration of the translation process can be found in Section 1 in Multimedia Appendix 1.

Initially, the stimulus material consisted of 10 unique fictitious patient messages, each paired with 10 LLM-generated responses and 10 human-generated responses, totaling 20 message/response pairs. Pilot testing indicated that this resulted in an excessively time-consuming survey. Therefore, 5 fictitious patient messages were randomly selected, each paired with 1 human-generated and 1 LLM-generated response. This resulted in a final stimulus set of 10 message/response pairs, all available in Section 2 in Multimedia Appendix 1.

#### Fictitious Patient Messages

The fictitious patient messages were written by 3 of the authors (TTL, LF, and HFP), who were all experienced with both face-to-face cognitive behavioral therapy and iCBT for FSD. The messages were designed to resemble the type of messages encountered within an ongoing randomized controlled trial (RCT), testing the iCBT treatment “One Step at a Time” for FSD [38]. First, authors constructed 5 fictitious patient profiles detailing central demographic and FSD characteristics. They then wrote 5 fictitious messages based on these profiles. Messages were written as authentically as possible, leaving in typing errors, abbreviations, idioms, and idiosyncratic language, and were aligned with the treatment principles of “One Step at a Time.” Messages had to be between 400 and 800 characters to ensure they aligned with the average length of patient messages observed in the RCT and were not excessively long [38].

#### Human Therapist–Generated Responses

To generate human therapist responses, 4 clinical psychologists at the Department of Functional Disorders, Aarhus University Hospital, all with experience as iCBT therapists in “One Step at a Time” [38], were invited to individually write responses to the fictitious patient messages. The group comprised 2 senior therapists, who were authorized clinical psychologists in Denmark with several years of experience treating FSD, and 2 junior therapists, who had treated patients with FSD within “One Step at a Time” but had less experience in other therapeutic contexts and were not yet authorized. Across the group, therapists had a median of 12 (range 4-13) years of clinical experience. The 10 messages were randomly distributed among the 4 therapists, with 2 therapists writing 3 responses each and 2 writing 2 each. Each therapist was provided with a brief instruction and an abbreviated overview of the treatment content of “One Step at a Time,” identical to the material used to prompt the LLM. They were instructed to write responses of about 800-1200 characters, emulating the average length of therapist responses in the RCT, and were allowed a maximum of 30 minutes per message, emulating the time pressure that characterizes routine clinical iCBT. In practice, the therapists used a median of 18 (range 15-25) minutes per message and produced responses with a median length of 1587 (range 1153-1968) characters.

#### LLM-Generated Responses

To emulate real-world clinical contexts, where iCBT communication is to be considered and treated as highly sensitive health data, all LLM responses in this study were generated using a locally hosted open-weight model providing full privacy and control over data flows. To do this, the Llama 3.1 70B model, quantized to 4-bit precision (IQ4_XS, GGUF format), was used via the llama.cpp inference engine. Inference was performed on 2 NVIDIA RTX 4090 GPUs (temperature=0.8; top_k=40; top_p=0.95; min_p=0.05).

A prompting template (available in Section 3 in Multimedia Appendix 1), developed by the authors, was used to generate LLM responses. The template consisted of 4 components: (1) the study context and instructions for the LLM, (2) an abbreviated version of the “One Step at a Time” iCBT protocol (abbreviated because the full version exceeded the LLM’s context window), (3) basic patient demographic information, and (4) the patient’s message. The abbreviation of the treatment protocol was produced iteratively and reviewed by the authors who developed the “One Step at a Time” program to ensure that all central concepts and exercises were retained in brief form. The instructions invited the model to take on the role of the therapist, respond in a professional, empathic, and helpful way, base the response on the inserted treatment content, and write between 800 and 1200 characters. No example responses were included, and no fine-tuning or retrieval-augmented generation was used. The same template was used for all messages, with only the patient information and message varying. The template was refined iteratively against pilot outputs, with the author group adjusting successive drafts until responses were judged contextually appropriate, before being finalized for generation. For each response, the prompting template and the fictitious patient message were entered into the model. The first LLM output generated was used, regardless of content or quality. The median length of LLM-generated responses was 1674 (range 1410-1756) characters.

### Measures

The quality of iCBT responses was rated on a set of 5 quality dimensions. These were informed by the items used in previous studies [12,13,17] and frameworks for assessing treatment fidelity and good clinical practice in iCBT [38,39] (for items, see Section 4 in Multimedia Appendix 1).

Quality dimensions included *quality* (“How would you estimate the overall quality of the response?”; 1: very low to 5: very high), *helpfulness* (“How helpful would you rate the response to be for the patient?”; 1: definitely not helpful to 5: definitely helpful), *empathy* (“The response is empathetic” 1: strongly disagree to 5: strongly agree), *professionalism* (“The response is professional”; 1: strongly disagree to 5: strongly agree) and *protocol adherence* (“The information provided by the therapist aligns with the treatment protocol”; 1: strongly disagree to 5: strongly agree). These 5 dimensions were all assessed with 1 item, scored on a 5-point rating scale. In addition, a sixth binary item asked raters to guess the *response source* (“Who would you guess wrote the response?”; human therapist/AI). An open-ended text field was presented for each message/response pair, where raters could provide context for their response on the items.

An attention item was placed in every fourth message/response pair (eg, “To ensure you are paying attention, please select ‘Neither or’ for this item”). To support clinicians in assessing protocol adherence, a drop-down field could be opened displaying a brief description of the “One Step at a Time” treatment concept.

The initial descriptive information included the following variables: name, nationality, affiliation, professional background, status as clinically active (ordinal), experience as a psychotherapist (binary), experience treating FSD (binary), experience as an iCBT therapist (ordinal), total years of clinical experience (scale), self-rated qualifications as a rater (5-point rating scale).

### Procedure

The messages and responses were compiled into a digital survey on Aarhus University’s version of REDCap (version 15.5.36; Vanderbilt University) [40]. The survey presented 1 message/response pair per page, followed by the rating items in a single randomly generated order that was held constant across all participants. Preceding the message/response pairs, a brief introduction to the experiment was provided.

Once data collection started, a list of possibly eligible institutions and raters was compiled among the authors based on their network and knowledge of the field. The main author (TTL) reached out to everyone on the list with a standardized email, requesting eligible recipients to participate in the study and to share the invitation within their network. Further, an advertisement for the study was posted on the social media site LinkedIn.

To enter the study, raters followed an open link to the REDCap survey. Here, raters were informed about the experiment and its legal basis, provided consent, and answered the descriptive information questions. After this, all message/response pairs were displayed one by one in a randomized order, with instructions to rate the therapist response on the standardized quality criteria.

### Analysis

#### Quantitative Analysis

Given the presumed limited size of the population that was sampled from, a preregistered exploratory analysis strategy was used, focusing on interpreting mean differences and CI between LLM- and human-generated responses for each quality dimension.

For each quality dimension, an individual mean score was calculated per rater by averaging their ratings across the 5 LLM-generated and 5 human-generated responses, respectively. This yielded 2 individual means per rater per dimension: 1 for LLM-generated and 1 for human-generated responses. These individual means were then averaged across all raters to produce grand means for each quality dimension separately for LLM- and human-generated responses. The grand means and their CIs were compared to assess overall differences between LLM- and human-generated responses.

For the item regarding the ability of raters to identify the source of each response (human/AI), a mean correct classification rate was calculated for each individual across all messages/response pairs. This was furthermore combined into a grand mean representing the overall correct classification rate for human and LLM-generated responses, respectively.

For all items, exploratory dependent *t* tests were conducted comparing grand means. These were, however, interpreted cautiously given the analytical strategy’s focus on descriptive analysis and omission of a priori statistical power calculations.

Subgroup analyses were conducted examining whether the difference in grand means were affected by rater characteristics (*Currently clinically active* [yes/no]; *Clinical experience with FSD* [yes/no]; *Clinical experience with iCBT* [with FSD/with other conditions/no]; *Profession* [medical doctor/psychologist]; *self-assessed rating qualifications* [qualified/not-qualified]; *seniority* [junior/senior]; *experience as a therapist* [yes/no]). For binary variables, this was done by calculating individual means for each level of the subgroup variables. Nonbinary variables were dichotomized: self-assessed rating qualifications were dichotomized by splitting the 5-point rating scale into qualified (“qualified/very qualified”) and nonqualified individuals (“definitely not qualified/not qualified/somewhat qualified”). Clinical experience was dichotomized by splitting experienced (10 or more years of self-reported clinical experience) from inexperienced (less than 10 years).

Raters who did not adhere to the eligibility criteria were excluded. To be eligible for analysis, each rater was required to have rated 3 or more message/response pairs for both LLM and human-generated responses. The 4 clinicians who had authored the human-generated responses all participated in the study. The quality rating of their own responses and the corresponding LLM-generated response were excluded to circumvent self-rating bias. Raters who failed any attention checks were excluded from analysis to avoid inattentive responding compromising data quality.

#### Analysis of Open-Ended Text Responses

To provide further context for the interpretation of the quantitative data, open-ended text responses were analyzed using descriptive thematic content analysis [41]. Analysis involved iterative cycles of familiarization, coding, generation of themes, reviewing themes, elaborating themes, and reporting findings.

For coding, the free QDAcity (version 1.4.4 software [42]) was used. In a single shared document with a shared pool of codes, each message/response pair was set up with all associated open-ended text responses below. Two researchers (TTL and ORC) independently coded the material. In the initial phase, each researcher coded half of the correspondence prima vista. In a subsequent phase, the researchers switched over to review each other’s coding. In the third phase, each coder reviewed the entire dataset, iteratively revising the codes until a sufficiently comprehensive coding structure was attained. All open-ended text responses were analyzed in the language they were written in.

Both coding researchers were naive to the dataset but had deep knowledge of the study and surrounding scientific field. Qualitative analysis was characterized by ongoing reflexive processes with iterative discussions on positionality, prior knowledge, and assumptions [43]. TTL has a background within research and clinical psychology with expertise in the clinical treatment of FSD and iCBT. ORC has a background in technoanthropology, working with iCBT as an IT consultant and project manager.

### Ethical Considerations

The study was assessed by the Ethics Committee of Central Denmark Region and found exempt from requiring ethical approval (case number 1-10-72-9-25). All raters provided informed consent prior to participation after being informed about the study’s purpose and legal basis. No patients were involved in the study. All rater data were collected and stored in accordance with institutional data protection guidelines using Aarhus University’s REDCap platform [40]. Rater identities are not disclosed in any published materials. Raters received no compensation for their participation.

## Results

### Overview

Across the inclusion period, a total of 71 possible raters accessed the survey, filling out the descriptive information form. Of these, 61 individuals had a full set of responses for 3 or more message/response pairs on both the LLM- and human-generated responses. Five raters were removed for failing the attention checks. One rater was removed for a suspicious reporting pattern (ie, having the same response for all items). One rater was removed for neither having clinical experience with FSD nor iCBT. This resulted in a total of 54 raters included for analysis. Of these, 53 had complete data for all message/response pairs, while one had missing values on the last 3 pairs. For descriptive information about included raters, see Table 1.

Five raters reported they were not currently clinically active, contrary to the eligibility criteria. These raters were, however, known to the authors to have relevant clinical experience. To retain as large a sample as possible, the authors chose to include these cases within the main analysis. To assess the effect of this choice, sensitivity analyses were conducted, removing these cases and rerunning all main analyses to see whether this changed the outcome.

**Table 1:** Descriptive information about included raters.

Characteristic				Value
Country, n (%)				
		Denmark		33 (61)
		Sweden		17 (31)
		Germany, Belgium, Finland		4 (7)
Professional background, n (%)				
		Psychologist		36 (66)
		Medical doctor		18 (33)
Clinically active (yes), n (%)				49 (90)
Experience as psychotherapist (yes), n (%)				43 (79)
Experience treating FSD^a^ (yes), n (%)				43 (79)
iCBT^b^ experience, n (%)				
	No experience			24 (44)
	Experience with iCBT for FSD			15 (27)
	Experience with iCBT for other conditions			15 (27)
Self-reported qualification to rate iCBT correspondences, n (%)				
			Definitely not qualified	1 (2)
			Not qualified	0 (0)
			Somewhat qualified	16 (29)
			Qualified	26 (48)
			Very Qualified	11 (20)
Age (years), mean (SD; range)				40 (9.0; 25-62)
Clinical experience (years), mean (SD; range)				12 (8.23; 1-34)

^a^FSD: functional somatic disorder.

^b^iCBT: internet-based cognitive behavioral therapy.

### Quantitative Results

**Figure 3:**
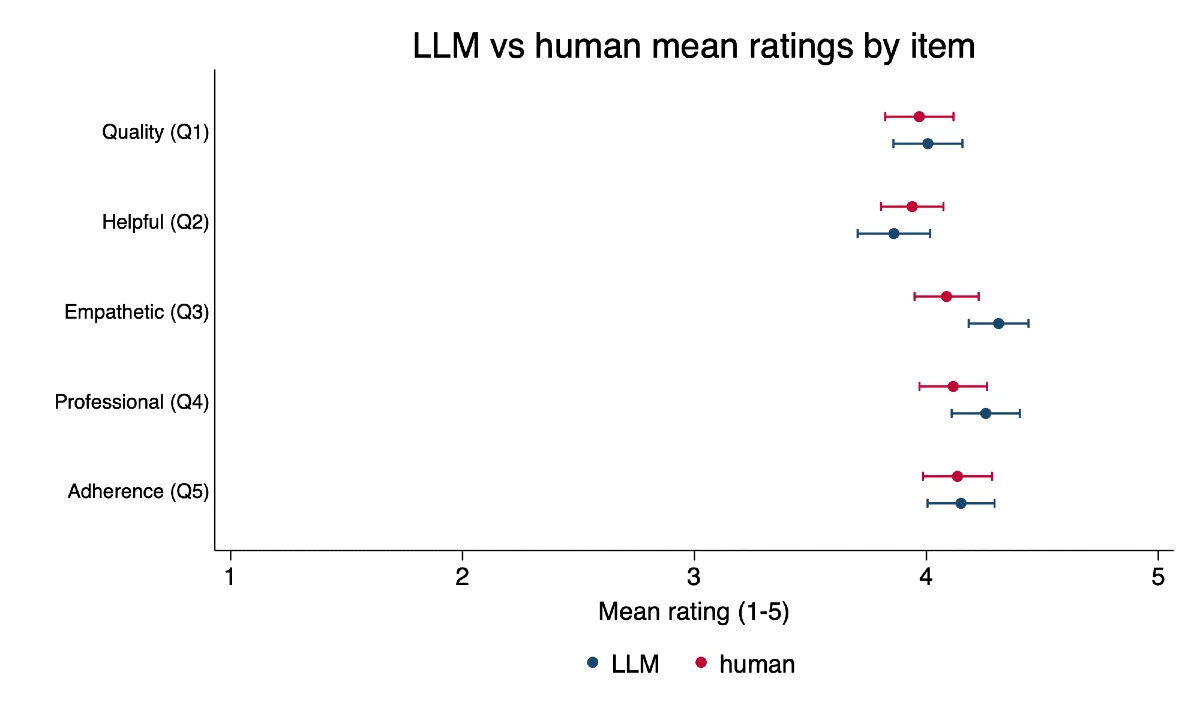
Mean rating (1-5) for large language model (LLM)– and human-generated responses across the 5 quality dimensions. Points show group means with 95% CIs. Higher scores indicate more favorable ratings.

As shown in Table 2, no large differences between LLM- and human-generated responses were observed across quality dimensions. The scale of difference varied from near-zero difference, for protocol adherence, to the LLM being rated as slightly higher on empathy than human-generated responses. The LLM was further rated slightly higher on professionalism and slightly lower on helpfulness. For an illustration of the scale of effects, see Figure 3. Across quality dimensions, both LLM- and human-generated responses tended to be rated with a mean score around 4, indicating that the quality of messages was generally perceived as above average in quality. Raters identified the source correctly more often than chance for both response types. Classification was substantially more accurate for human-generated than LLM-generated responses.

To explore the effect of rater characteristics, subgroup analyses were conducted for all moderator variables (see Figures a-g in Section 5 in Multimedia Appendix 1). Across quality dimensions, few moderator variables affected the rating of responses and the classification success rate of individuals. One notable variable was self-reported status as clinically active. A total of 5 individuals rated themselves as clinically inactive, and these tended to consistently favor LLM-generated responses more. The scale of difference was nevertheless small, in no case exceeding one point on the 5-point rating scale.

Sensitivity analysis was conducted to examine the effect of including the 5 individuals who rated themselves as currently not clinically active. Results are presented in Section 6 in Multimedia Appendix 1. Marginal differences were observed across quality dimensions, indicating little evidence that the main analysis was biased by the inclusion of individuals with prior, but no current, clinical activity.

**Table 2:** Mean rating scores for all items for LLM^a^ and human-generated responses.

Item	LLM^a^, mean (SD)	Human, mean (SD)	Δ^b^ (95% CI)	Cohen *d*	*P* value^c^
1. Quality	4.00 (0.54)	3.96 (0.53)	0.04 (–0.13 to 0.21)	0.06	.66
2. Helpfulness	3.85 (0.57)	3.93 (0.49)	–0.08 (–0.24 to 0.08)	0.13	.32
3. Empathy	4.31 (0.47)	4.08 (0.50)	0.22 (0.08 to 0.37)	0.42	<.001
4. Professionalism	4.25 (0.53)	4.11 (0.53)	0.14 (–0.08 to 0.29)	0.24	.08
5. Protocol adherence	4.13 (0.52)	4.13 (0.54)	0.01 (–0.11 to 0.14)	0.03	.81
6. Correct classification^d^	62.72 (21.30)	78.64 (19.65)	–16 (–21 to –11)	0.79	<.001

^a^LLM: large language model.

^b^Mean difference between LLM and human.

^c^Significance level of *t* test.

^d^Correct classification is reported as the mean percentage of responses correctly identified per rater. Values for this row, including Δ and the 95% CI, are expressed in percentage points rather than on the 1 to 5 rating scale.

### Results From Analysis of Open-Ended Text Responses

**Figure 4:**
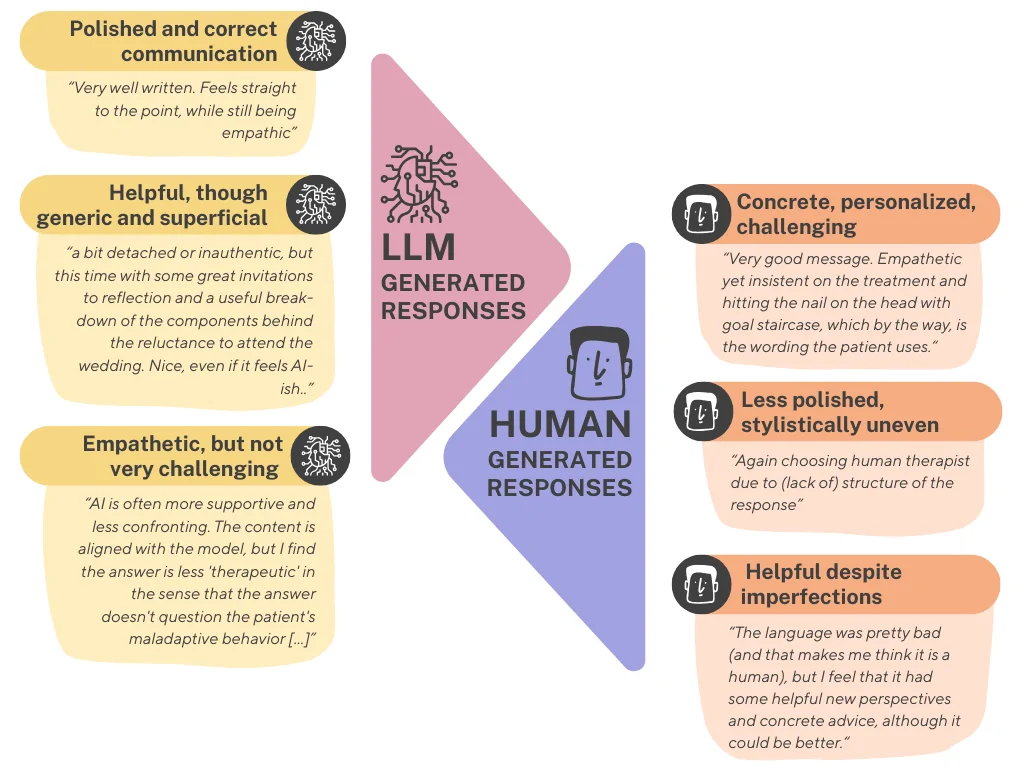
Findings from the descriptive thematic content analysis. Italicized text represents selected quotes from raters. LLM: large language model.

A subset of 30 (55%) raters wrote comments in one or more open-ended text fields, yielding 166 commented text fields, totaling more than 4900 words. The raters who responded to the open-ended text fields did not differ meaningfully from the full sample on demographic characteristics or the main outcome measures (Sections 7 and 8 in Multimedia Appendix 1). Findings from the analysis of the open-ended text fields, including selected quotes, are summarized in Figure 4. A codebook with codes and coding frequencies is available in Section 9 in Multimedia Appendix 1.

Two broad patterns were observed: LLM-generated responses tended to be characterized as well-written, structured, often inviting reflection and providing meaningful advice. At the same time, they were characterized as generic, impersonal, and decoupled from the message they were responding to. Many clearly described these replies as empathetic, although the empathy conveyed was at times experienced as excessive. This occasionally resulted in advice described as insufficiently challenging, potentially reinforcing avoidance behavior.

Descriptions of human-generated responses were weighted differently. These responses were often perceived as concretely helpful and personalized. Specifically, human-generated responses were often praised for having a good balance between being empathetic, directive, and challenging, providing concrete suggestions for making behavioral changes. The human-generated responses tended to be described as more unstructured, verbose, informal, or stylistically uneven, which was frequently provided as a reason for assuming it was written by a human. The less polished appearance of human-generated responses, however, did not appear to detract from the perceived usefulness of these messages.

## Discussion

### Principal Findings

This exploratory experimental study aimed at taking first steps toward assessing whether an LLM would be able to generate useful responses to fictitious patient messages in iCBT for FSD compared with human therapist responses. Clinician ratings indicated that LLM responses were broadly comparable to human responses across the 5 quality domains. The observed mean differences ranged from 0.01 to 0.22 on the 5-point scales and were considered marginal. These results should be interpreted descriptively as the analyses were exploratory and not powered for formal testing. While the numerical direction is consistent with the expectations for overall quality and empathy (H1 and H2), the differences were considered too small to be meaningful. Source identification exceeded chance in both cases, particularly for human-generated responses, which runs counter to the expectation of chance-level source identification (H3).

Qualitative findings further cast important nuance on the quantitative results: while LLM responses were described as polished and empathetic, the empathy conveyed was at times described as excessive and came at the expense of being appropriately challenging. Human-generated responses, by contrast, were seen as less polished but more concrete and personalized. Why the quantitative and qualitative findings diverged is not clear, and several explanations are possible. The single-item scales may have been too coarse to register the more specific shortcomings that raters articulated in the open-ended text fields. The divergence may also reflect how the comments were generated: only a select portion of raters wrote free-text comments, and raters may be more inclined to comment when they have a reservation than when they are satisfied, so the qualitative material may overrepresent critical observations relative to the overall ratings.

LLM-generated responses were classified correctly only slightly above chance, indicating that the output was fluent and contextually appropriate enough not to read consistently as LLM-generated, in contrast to human-generated responses, which raters identified more readily. The qualitative findings offer one way to understand this asymmetry: stylistic unevenness, informality, and idiosyncrasy were frequently cited in the open-ended text fields as reasons for attributing a response to a human, whereas LLM responses were described as polished and structured. Human-typical features may therefore have served as more diagnostic cues than the comparatively ambiguous polish of the LLM responses, since experienced therapists can also produce polished text.

Across subgroup analyses, rater characteristics had little bearing on the ratings, with no difference exceeding one point on the scale. The only notable tendency, that the 5 clinically inactive raters favored LLM-generated responses slightly more, rested on too small a subgroup to interpret with confidence, and a sensitivity analysis excluding these raters left the overall pattern essentially unchanged.

Results are largely in line with previous studies using similar experimental rating designs, where models have been found to be comparable to human therapist responses, in certain cases, conveying more empathy and compassion [12-17,32]. This study extends this literature by being among the first to evaluate LLM-generated responses within an iCBT setting where a concrete pathway to clinical implementation exists. It further strengthens the evidence base by relying on raters with domain-specific clinical expertise. Given the deliberately constrained experimental rating design, however, the findings do not speak directly to clinical practice, but provide an initial feasibility check and represent a first step toward more naturalistic, clinically embedded studies.

Given the risks and legal constraints involved in LLM integration into mental health care, the most immediately viable use case for LLM technology may be as a support tool for therapists [20,22,23]. This, however, does not diminish the potential of LLMs within iCBT. Lack of qualified mental health care professionals is a central driver of unmet mental health treatment needs worldwide [1-3]. This is also the case in iCBT, where the reliance on human therapists limits scalability. In this study, for instance, therapists indicated using upwards of 15-25 minutes per message. With multiple active patients, the cumulative workload becomes substantial.

In the most favorable framing, LLM implementation could markedly improve clinician workflow efficiency, without compromising clinical outcomes. One experimental study of professional writing tasks suggests that LLM assistance can substantially reduce task completion time [44]. If similar gains extend to iCBT, this could improve scalability, letting each therapist support more patients without compromising outcomes, or, alternatively, reduce the workload per message so therapists can support patients more closely, potentially strengthening adherence and thereby outcomes.

Implementing LLM-based automation is no simple task, however, and whether these technologies deliver on their promise in a complex clinical reality remains unclear [45]. Few studies exist on this topic [46], but one clinical implementation study, examining implementation of LLM drafting in a medical context, found no reduction in time spent per message [47]. This may reflect the complexity of automation and decision support tools: while a digital tool may solve a problem efficiently (eg, drafting an iCBT response), it can also generate other types of tasks (eg, writing prompts and reading and revising drafts) which offset initial efficiency gains [45,48,49]. This dynamic makes actual efficiency gains of LLM technologies difficult to predict and underscores that investments in automation tools should rest on careful analysis of how digital tools interact with existing workflows.

Furthermore, it is unclear how the implementation would affect the experience of therapists. Automating message drafting may be experienced as a relief, reducing cognitive load and freeing therapists to focus on more meaningful clinical tasks. Emerging evidence on AI support tool implementation, for instance, indicates that it may improve the feeling of autonomy, reduce cognitive load and burnout [47,50,51]. However, gains in therapist efficiency could also raise management expectations about patient caseloads, potentially offsetting positive effects on the occupational health of therapists [49]. At the same time, it could create the impression among therapists that core professional competencies are being outsourced, reducing therapists to peripheral auditors rather than central actors in the therapeutic process [49]. This could negatively affect iCBT therapists’ willingness to adopt LLM-based tools and lead to shifts in their work satisfaction, commitment, and professional identity. Prolonged use of LLM-based tools may also put clinicians at risk of automation bias, where LLM-generated suggestions are accepted without sufficient critical evaluation [52,53], and may lead to deskilling, where clinicians’ capacity to independently formulate therapeutic responses gradually deteriorates [49,54]. Over time, this could shift therapists’ sense of what constitutes good clinical practice and erode the distinctly human dimensions of therapeutic work.

Realizing the potential of LLMs in iCBT will therefore require a carefully researched approach to implementation that accounts for these subtle but consequential effects on clinical practice. Central to this may be framing LLM tools as a means of supporting and enhancing clinical competencies rather than replacing them, since research suggests that passively relying on AI-generated content erodes workers’ self-efficacy, ownership, and sense of meaning, whereas actively engaging with and revising output largely preserves them [55]. Practically, this could mean enabling clinicians to prompt the model themselves rather than only receiving drafts, so that clinical judgment is actively applied. It may also be central to provide them with sufficient LLM literacy, establishing local guidelines for how to use LLM systems and providing access to regular supervision to understand and safely use the systems they work with [49]. Developing these systems in collaboration with clinicians and facilitating local configurability may also ensure they optimally support real workflows and foster a sense of shared ownership among users.

Another important aspect of LLM implementation is the experience of patients receiving LLM-augmented therapist guidance. The therapeutic relationship is often considered a central ingredient of change in psychotherapy [56], including digital interventions [57,58]. If patients are aware that therapist responses are partially generated with LLM assistance, the empathy conveyed in iCBT communication may risk being perceived as less genuine or credible [59]. This is supported by certain studies indicating that AI-labeled responses are viewed as less trustworthy and of lower quality than human-labeled responses [16,32,60]. Within mental health service delivery, users also generally report a preference for a human-in-the-loop approach to AI integration [61]. Careful integration of LLM features into iCBT workflows may therefore be important to preserve the core relational elements of therapist guidance.

Beyond the various challenges relating to clinical implementation, integrating LLM support into iCBT also poses a technical challenge. It is possible to imagine several different specific functions that could be supported by an LLM in iCBT: summarizing courses of treatment, drafting responses, and providing intervention suggestions. Most pertinent for this paper is drafting support. This would entail installing a feature in existing iCBT platforms which, when prompted by a therapist, would package relevant clinical context (eg, treatment program details and patient data), send it to the LLM with an instruction prompt, and return a draft for the therapist to review and send. Such systems would also require operational oversight, such as automated monitoring of LLM outputs to sustain acceptable performance and guard against model drift [62].

Given the sensitive, clinical nature of iCBT correspondences, LLMs would most likely require local hosting to ensure full control over dataflows. LLMs, however, require substantial computational resources, creating potential cost barriers for many clinical organizations [63,64]. Notably, within this study, a 70B parameter LLM was used with long but simple prompt instructions, yet still seems to generate response drafts that appear relevant and feasible in the context. This is interesting given the common assumption that LLMs require context-specific optimization (eg, fine-tuning or retrieval-augmented generation) to function effectively in specialized domains [8,64]. While some studies suggest that fine-tuning to mental health contexts can improve performance [65,66], others indicate that it may not yield consistent gains and can even degrade output quality, with performance depending more on base model scale and architecture [67]. A related consideration regards cultural and linguistic optimization. LLMs generally perform best in high-resource languages such as English and may have impaired performance in smaller languages (eg, Danish) [35,36], which has also been indicated in a mental health context [37]. Linguistic and cultural performance in low-resource languages could potentially be improved by using language-adapted foundational models [68].

Given that each optimization strategy entails distinct resource investments, future work should systematically examine whether the resulting performance gains justify the costs relative to using general-purpose base models. This could inform evidence-based decisions about how LLMs can be most effectively optimized and implemented into iCBT workflows.

### Limitations

While this study provides initial insight into the feasibility of applying LLMs in iCBT, current results and conclusions should be interpreted in the light of several important methodological limitations:

First, this study was conducted as a highly controlled clinician rating pilot experiment that had to balance ecological validity and generalizability to real iCBT contexts against the need to generate rigorous, standardized data on response quality. It also had to accommodate the need to recruit from a highly limited population of qualified clinicians, each with limited time to allocate to responding to surveys. These constraints required methodological compromises that limit generalizability to real-world iCBT workflows: ratings of both human- and LLM-generated responses were based only on simulated single-turn iCBT exchanges and basic demographic information. This should be considered quite different from routine iCBT, where a therapist typically has access to patient records, prior platform interactions, and video or telephone consultations, and where exchanges unfold over multiple turns rather than one. The use of single-turn exchanges is a particularly important limitation, given the tendency for LLM performance to degrade substantially over multiturn exchanges [62]. Nevertheless, this approach is consistent with previously conducted comparable studies [12,16,17] and was the only feasible option given the study’s exploratory scope.

Second, the experiment was conducted in English, as this was assumed to be the fairest test of the LLM’s capabilities and allowed for international recruitment of raters. The human therapists and researchers were, however, only clinically proficient in Danish, and the patient messages and human-generated responses were therefore written in Danish and translated into English. Although translation was conducted by a qualified translator, reviewed by the original authors, and checked by a native English speaker, it may still have subtly altered the tone, style, and feel of the message. Importantly, while the patient messages were translated for both conditions, only the human-generated responses were themselves produced in Danish and translated, while the LLM generated its responses directly in English. Any loss of nuance from translating the responses could therefore have affected the 2 response types differently and cannot be ruled out as a contributor to the observed variability in response characteristics.

Third, the choice to include only qualified clinician raters meant that the population that could be sampled from was small. This gave rise to a relatively small sample size, barring adequate use of inferential statistics. The sample size was nevertheless not much different from other studies using qualified raters [13,16,17] and compared to similar studies using medical domain expert raters [69,70]. The use of experienced clinician raters also means that no patients were included, which means it remains unclear whether patients would perceive the LLM-generated messages similarly to the experienced clinician raters. Further, raters were recruited primarily through the authors’ professional networks, which may have introduced selection bias. Clinicians reached this way may hold similar attitudes toward digital technologies or LLMs, whether explicit or implicit, that are not representative of the broader population, potentially affecting their ratings and limiting the diversity of perspectives in the sample. Relatedly, rater attitudes toward AI were not measured in this study, making it difficult to discern whether they moderated the ratings.

Fourth, each of the 5 quality dimensions was assessed with a single item rather than a validated multi-item scale. This was a deliberate decision to keep the survey short enough for time-constrained clinicians to complete, and a consistent approach used in comparable rating studies [13,16,17]. Single items nevertheless capture each construct more coarsely than multi-item scales and provide no estimate of internal consistency, which limits the measurement validity and reliability of the ratings.

Fifth, the need to limit survey length meant only 5 patient messages, each paired with 1 human- and 1 LLM-generated response, were evaluated. As a pilot aimed at establishing the direction and magnitude of effects rather than a definitive estimate, detailed ratings from a sufficient number of raters were prioritized over a broad stimulus set, consistent with previous similar studies [13,16]. Five messages, however, cannot capture the range of clinical situations, registers, and communicative challenges encountered in iCBT, and the messages sampled may be ones the model handles relatively well. Larger, more varied stimulus sets are therefore needed and could reveal weaker or less reliable performance.

Sixth, the study evaluated a single model (Llama 3.1 70B) in a single configuration, using only the first generated response for each message. Because LLM output is probabilistic, this single draw does not capture the model’s variability, and performance may vary across models and configurations, though the relatively small, nonoptimized model used here represents a conservative baseline that larger or more recent models would generally not be expected to underperform. Using the first output avoided researcher curation but means the rated responses may not reflect the model’s best performance. Comparison across models, configurations, and repeated samples is an important direction for future work.

Seventh, the study assessed perceived response quality but included no dedicated clinical safety assessment. Professionalism and protocol adherence may capture some safety-relevant aspects but cannot substitute for systematic evaluation of clinical risk, such as inappropriate reassurance, reinforcement of avoidance, inadequate handling of suicidality, or advice conflicting with treatment principles.

### Implications and Future Studies

Findings from this study indicate that LLMs could potentially be implemented as a useful support tool for therapists in iCBT. However, further studies, gradually moving from small-scale experiments to clinical studies, are needed before responsible implementation can be realized [24].

To improve the ecological validity of the findings, these findings should be replicated using a larger and more varied set of real-world iCBT patient messages rather than constructed stimuli, extended to multiturn exchanges, and provide therapists access to much more contextual information than just the isolated demographic characteristics and message/response pair. Future research efforts should also adopt validated multi-item scales, which include assessments of perceived safety and avoid the present translation asymmetry by conducting the comparison natively in one language. Such studies could also include iCBT patients as raters, thereby capturing end users’ perceptions of usefulness instead of only including expert raters’ perspectives. Recruitment should also rely less on the researchers’ professional networks and aim for a more representative sample, and raters’ attitudes toward AI should be assessed and accounted for in the analysis. Finally, research should examine how the introduction of LLM-assisted messaging may influence the therapeutic relationship in iCBT.

To guide effective implementation, studies could also explore how the introduction of LLM technologies affects the iCBT workflow for therapists, both on a practical (eg, time savings and additional work processes) and an experiential level (eg, changes to professional identity and work satisfaction). Research should also examine whether prolonged use of LLM-based tools introduces risks of automation bias or clinical deskilling among iCBT therapists. Further, studies could explore how LLM performance in an iCBT context is affected by different optimization strategies and whether the resulting gains justify the associated costs (eg, retrieval-augmented generation, fine-tuning, and linguistic/cultural adaptation).

Ultimately, LLM-based tools should be tested within clinical iCBT trials to assess their impact on treatment outcomes and cost-effectiveness, while also monitoring for unexpected downstream effects on both therapist and patient experience.

### Conclusions

This study examined to what extent LLMs can generate meaningful responses to patient messages in iCBT for FSD. Across 5 therapeutic quality domains, experienced clinician raters judged the model’s responses to be of broadly comparable quality to those written by experienced human therapists. Qualitative findings provided further context, indicating that while LLM outputs were perceived as polished and well-formulated, they were also at times experienced as generic, excessively empathetic, and insufficiently challenging. These findings provide initial indications of the feasibility of using LLMs as a therapist-support tool within iCBT workflows. Future research should examine how LLM-based tools can be effectively integrated into iCBT workflows and whether their use translates into tangible clinical and organizational benefits.

## Appendix

Multimedia Appendix 1 Stimulus materials, data collection, and analyses.

## Abbreviations

FSD: functional somatic disorder
iCBT: internet-based cognitive behavioral therapy
LLM: large language model
OSF: Open Science Framework
RCT: randomized controlled trial

## Data Availability

Anonymized data, code, and other materials can be made available upon contact with the corresponding author.
