# Supplementary material for: Large Language Models as a New Tool for Therapists in Internet-Based Cognitive Behavioral Therapy: Blinded Clinician Rating Pilot Experiment
Source: JMIR Ment Health. 2026 Aug 26;13:e96835. doi: 10.2196/96835 (PMC13559149; doi:10.2196/96835)
Supplement: Supplementary file 1 [file mental_v13i1e96835_app1.docx]

# **Supplementary materials:** Large language models as a new tool for therapists in internet-based cognitive behavioral therapy? A blinded clinician rating pilot experiment

## Authors

Thomas Tandrup Lamm^1,2^

(Corresponding Author, THLAMM@rm.dk , +45 61 14 70 69)

<https://orcid.org/0009-0001-4335-5640>

Arthur Bran Herbener ^3^

<https://orcid.org/0009-0009-0585-2599>

Oliver Rønn Christensen ^2^

<https://orcid.org/0000-0002-3964-5514>

Malene Flensborg Damholdt^3^

<https://orcid.org/0000-0001-9352-6703>

Kaare Bro Wellnitz ^2^

<https://orcid.org/0000-0001-5882-5118>

Heidi Frølund Pedersen ^1,2^

<https://orcid.org/0000-0002-8612-5466>

Lisbeth Frostholm ^1,2^

<https://orcid.org/0000-0002-9683-7416>

1, Department of Clinical Medicine, Aarhus University, Palle Juul-Jensens Boulevard 11, 8200 Aarhus N, Aarhus, Denmark

2, Department of Functional Disorders, Aarhus University Hospital, Palle Juul-Jensens Boulevard 11, 8200 Aarhus N, Denmark

3, Department of Psychology and Behavioral Sciences, Aarhus University, Bartholins Allé 11, 8000 Aarhus C, Denmark

**Contents of supplementary materials:**

- Section 1: Illustration of the generation of stimulus materials
- Section 2: Fictitious patient-therapist correspondances used as stimulus material
- Section 3: Prompting template
- Section 4: Items used for data collection
- Section 5: Results of subgroup analyses
- Section 6: Sensitivity Analysis
- Section 7: Characteristics of responders to open-ended text fields
- Section 8: Ratings on the main outcome variables of responders to the qualitative analysis
- Section 9: Codebook from qualitative analysis

## Section 1: Illustration of the generation of stimulus materials

## Section 2: Fictitious patient-therapist correspondances used as stimulus material

*Each title contains two numbers. The first indicates the item's placement in the sequence shown to participants. The second identifies the numbering of the patient messages. Each patient messages appears twice, once with an LLM response and once with a human therapist response, as marked in the final parentheses.*

### Message-answer pair 1_9 (LLM)

Patient Message

Dear Helene

It has been a really tough week. I am sleeping poorly and I am very tired. The pains are also quite bad at the moment, and I have therefore not had the energy to look at the program this week. Can I have a week more to look at module 5? I find it difficult to find time for everything. Last weekend my dad fell in the bathroom in the middle of the night and was unable to get up on his own, so my mother called me for help, and I been with thdm most days to help with food and shoppin although I am really not up to it. It affects my pains and I just get more tired. I really want to be more active in the program, but I am just not really coping. I hope you understand.

Therapist response

Dear Pernille,

I'm so sorry to hear that you're having a tough week. It's completely understandable that you're feeling overwhelmed and struggling to keep up with the program. Taking care of your parents is a big responsibility, and it's admirable that you're there for them, even when you're not feeling your best.

First of all, please don't worry about falling behind with the program. You can take all the time you need, and we can adjust the pace to suit your needs. I'd be happy to give you an extra week to work on module 5. In fact, it might be helpful to take a break and focus on your own well-being for now.

It's great that you're recognizing the impact that caregiving is having on your symptoms and energy levels. That's a big step in taking care of yourself. Remember that self-compassion is an important part of this program, and it's okay to prioritize your own needs when you need to.

Rather than pushing yourself to do more, let's focus on finding ways to manage your symptoms and conserve your energy. Have you tried using some of the techniques we've covered in the program, such as the STOP exercise or attention training, to help you cope with the stress and fatigue? These might be helpful in managing your symptoms and finding some calm in the midst of all this.

Remember, you're not alone in this. I'm here to support you, and we can work together to find ways to make this program work for you, even on the tough weeks.

Take care of yourself, Pernille, and don't hesitate to reach out if you need anything.

Best regards,

Helene

### Message-answer pair 9_9 (Human Therapist)

Patient Message

Dear Helene

It has been a really tough week. I am sleeping poorly and I am very tired. The pains are also quite bad at the moment, and I have therefore not had the energy to look at the program this week. Can I have a week more to look at module 5? I find it difficult to find time for everything. Last weekend my dad fell in the bathroom in the middle of the night and was unable to get up on his own, so my mother called me for help, and I been with thdm most days to help with food and shoppin although I am really not up to it. It affects my pains and I just get more tired. I really want to be more active in the program, but I am just not really coping. I hope you understand.

Therapist response

Dear Pernille,

Thank you for writing. It makes perfect sense with your father's fall and the increase fatigue and more pains than before. In that way, it makes perfect sense that there has been no check-in to the program and that you have not worked further. I think it makes perfect sense to look into module 5 again next week, it is about avoidance behavior.

When unforeseen things happen, like the situation you were in last week when your father fell and you feel an urge to help, the consequence is unfortunately that you pay the bill for how your body reacts afterwards. You write it yourself, "this is actually too much for me" so you are actually fully aware of it now.. On the one hand you probably want to help and be there for them, on the other hand you feel the consequences. In the situation you end forgetting to notice and set boundaries so it ends up in the "boom and bust" pattern.

You may need to ask yourself if you can help less than you have done in the last week eg by ordering groceries for them online instead of doing the shopping yourself, or getting some other relatives involved a little more, so that you can help without putting yourself on hold helping with creative solutions instead of overusing yourself. Does it make sense that you could also use the principles from your treatment here to sign in and out more so that there is room for you and your treatment?

It doesn't have to be unloving or cold, but more compassionate towards both yourself and them, even though it may be difficult and unfamiliar at first.

I hope that makes sense?

Helene

### Message-answer pair 3_7 (LLM)

Patient Message

Dear Anna,

I can tell that you really think that I have too much going on, and others have said the same thing. But honestly, I don't feel ready to change anything right now. Deep down, I think that if I can't handle it all, it means I'm weak, and I am of course privileged compared with so many others. At the same time, I do understand your point that if I don't make some changes, then nothing is likely to improve.

I was just hoping that this situation could be solved differently because it is REALLY difficult. I can see how the goal staircases might be helpful for some people (me?), however, I just don't feel like using it, actually think it is a little embarrassing (and also embarrassing to tell you that I think that it is embarrassing,). Okay so now I don't exactly know what to ask you but at least you know how I feel right now.

Best regards,

Mette

Therapist response

Dear Mette,

Thank you for sharing your honest feelings with me. It takes a lot of courage to admit when we're struggling, and I appreciate your willingness to be open with me.

Firstly, I want to assure you that it's completely normal to feel overwhelmed and unsure about making changes. It's understandable that you might feel like you're not ready to let go of certain responsibilities or commitments, especially if you've always prided yourself on being able to handle everything.

However, I want to gently challenge the idea that not being able to handle everything means you're weak. In reality, recognizing your limits and asking for help is a sign of strength, not weakness. It's okay to acknowledge that you can't do it all, and that's where we can work together to find ways to prioritize and manage your tasks more effectively.

Regarding the goal ladder, I understand your initial hesitation. It's true that it might feel a bit awkward or embarrassing at first, but trust me, it's a powerful tool that can help you break down your goals into smaller, manageable steps. It's not about being perfect; it's about making progress and celebrating small wins along the way.

Remember, we're not trying to add more to your plate; we're trying to help you find ways to simplify and prioritize what's truly important to you. Your values and goals are what drive this process, and I'm here to support you every step of the way.

If you're willing, I'd like to explore some small, incremental changes that might help you feel more in control of your situation. We can start with tiny steps, and I'll be here to guide and encourage you along the way.

What do you think? Is there one small area where you might be willing to start making some changes?

Best regards,

Anna

### Message-answer pair 7_7 (Human Therapist)

Patient Message

Dear Anna,

I can tell that you really think that I have too much going on, and others have said the same thing. But honestly, I don't feel ready to change anything right now. Deep down, I think that if I can't handle it all, it means I'm weak, and I am of course privileged compared with so many others. At the same time, I do understand your point that if I don't make some changes, then nothing is likely to improve.

I was just hoping that this situation could be solved differently because it is REALLY difficult. I can see how the goal staircases might be helpful for some people (me?), however, I just don't feel like using it, actually think it is a little embarrassing (and also embarrassing to tell you that I think that it is embarrassing,). Okay so now I don't exactly know what to ask you but at least you know how I feel right now.

Best regards,

Mette

Therapist response

Dear Mette,

Thank you for your message and thank you for sharing how you are feeling. Being in treatment here is hard work, and you are definitely not alone in thinking it is a very difficult process. I can clearly recognize what you write from others I have in treatment. Especially this sense of feeling that you are weak for not being able to handle everything that you used to be able to. For many, having to change one's life comes with a great sorrow because it feels like changing who you are. I wonder how it feels for you? It really is a process, and it is completely okay to dwell a bit around the fact that it is difficult.

When you write to me, I also get the sense that you can maybe be a bit hard on yourself at times? Most people tend to be quite hard on themselves, and demand things from themselves that they would never demand from others. And tend to say things to themselves that they would never say to a good friend who was going through a hard time. There will be much more about what we call self-compassion later in the treatment, but it can be useful to already now start reflecting on whether you have a tendency to be hard on yourself, and what role it might play in the course of this treatment.

It's good that you're writing to me about the goal staircase. It's a fundamental element in the treatment, but as a tool it obviously needs to be adapted to you so it makes sense for you to work with it. Could you perhaps write a little more about what you find embarrassing about working with the goal staircase? We can put whatever you want into the goal staircase, and I'd really like to help you find out what would make sense for you to work with, so that it feels like something you can see yourself in.

Thanks again for sharing! Write to me if you need us to go through some of this over the phone.

Kind regards

Anna

### Message-answer pair 2_5 (LLM)

Patient Message

hi,

yes i usually manage to heolp elp out with the chores at home, but i just get so tired. I end up forcin myself just to avoid feeling guilt and my parents like god why are you pushing yourself? just let us help you. They're ofc trying to be kind, but I can also tell they're a bit like ughhh and maybe even a bit annoyed :(

Its just the program brings up some pretty difficult thoughts about the future cus if I cant even help in the kitchn how would I ever ever get an education and sometimes I get really panicky at night, when im lying with the headache

That's probably also why I haven't been using it the past cuple of weeks, ive just found it hard to get started with it, because honestly, I dont really want to think about it.

Therapist response

Hi Sara,

I completely understand why you'd feel overwhelmed and anxious about your situation. It's great that you're acknowledging your feelings and concerns, and I'm here to support you.

Firstly, I want to acknowledge your efforts in helping out with chores at home. It takes a lot of courage to push through fatigue and discomfort, and I can see that you're trying to maintain some sense of control and independence.

It's also understandable that your parents might be frustrated or worried about you, but it's essential to remember that they're coming from a place of love and concern. Their reactions might be a reflection of their own fears and uncertainties about your future, rather than a personal attack on you.

Regarding the program, I get why it can be daunting to confront difficult thoughts about your future. It's natural to feel anxious about not being able to pursue your education or achieve your goals. However, avoiding the program might not be the solution. Instead, let's work together to break down these overwhelming thoughts into smaller, manageable chunks.

One step at a time, we can explore ways to help you build resilience, develop coping strategies, and work towards your goals. It's okay to take things slowly and focus on your well-being right now. Remember, your health and happiness are crucial for achieving your long-term goals.

Let's start by identifying some small, achievable steps you can take this week. What do you think might help you feel more in control or confident? Is there something specific you'd like to work on, like managing your fatigue or finding ways to cope with anxiety at night?

Remember, I'm here to support you, and we'll work together to find ways to make progress, no matter how small.

### Message-answer pair 4_5 (Human Therapist)

Patient Message

hi,

yes i usually manage to heolp elp out with the chores at home, but i just get so tired. I end up forcin myself just to avoid feeling guilt and my parents like god why are you pushing yourself? just let us help you. They're ofc trying to be kind, but I can also tell they're a bit like ughhh and maybe even a bit annoyed :(

Its just the program brings up some pretty difficult thoughts about the future cus if I cant even help in the kitchn how would I ever ever get an education and sometimes I get really panicky at night, when im lying with the headache

That's probably also why I haven't been using it the past cuple of weeks, ive just found it hard to get started with it, because honestly, I dont really want to think about it.

Therapist response

Hi Sara,

Thank you for your message.

It sounds like you're getting really frustrated and almost end up helping at home to keep the guilty conscience at bay.

You also write that thoughts about the future come up and that the thoughts become very negative. You equating what you can do now with what you should be able to do in the future, e.g. with getting an education etc. When you then lie down at night with a headache you experience becoming completely panicked, and you get further and further away from the program in the last few weeks. It sounds like you are in a difficult place right now, where the small steps forward and stability are difficult to be in, that you become a little discouraged, is that something you can recognize?

You should know that you are not alone in getting frustrated, because the desire to be able to do more is great and therefore it is difficult not to think that things should go faster or further forward, it is exactly called one step at a time because it takes time and is a process that does not produce results as quickly as you might want.

I notice that there are a lot of thoughts, you also mention symptoms and frustration, wonder if it notices that you are being hard on yourself. I wonder what would happen if you tried to meet yourself as if you were good friend you were supposed to help.

I would really like to help you get started again, maybe we should take a step back to the last thing you did, would you be up for that?

Best regards

Lea

### Message-answer pair 18_4 (LLM)

Patient Message

Dear Jacob

I have now had a talk with my superior about having more downtime, and he seemed fine with me taking more breaks. So I will try that.

I am, however, really stressed about going to a wedding this Sunday. I already feel that it is going to be a real shitshow. I am already wasted after my work week and just know that the next week will be even worse when I don't get a chance to recover at the weekend.

Im considering to just pull out in order to be able to focus on one thing at the time. This will however mean another fight with Mathilde who hates that we again and again have to disappoint due to my tiredness. Maybe I should just go and then try to take some breaks while we are there.

Best regards

Gustav

Therapist response

Dear Gustav,

Thank you for sharing your concerns about the upcoming wedding and how it's affecting your stress levels. It's completely understandable that you're feeling overwhelmed, especially after a long work week.

Firstly, congratulations on taking the step to talk to your superior about needing more downtime. That takes a lot of courage, and it's great that they're open to accommodating your needs.

Regarding the wedding, I can see why you're feeling anxious about it. It's natural to feel like you're already running on empty, and the thought of a big social event can be daunting. Your instinct to prioritize your well-being and consider pulling out is valid. However, I also understand your concerns about disappointing Mathilde and the potential conflict that might arise.

Rather than making a hasty decision, let's break it down together. What are your biggest concerns about attending the wedding? Is it the social aspect, the noise, the crowds, or something else? Are there any specific aspects of the event that you're dreading?

Also, what do you think would happen if you did take breaks during the wedding? Would that help you feel more manageable, or would you still feel like you're stuck in a situation that's draining your energy?

Lastly, have you considered talking to Mathilde about your concerns and seeing if you can find a compromise together? Perhaps there's a way to attend the wedding that works for both of you, or maybe there's an alternative solution that you haven't thought of yet.

Remember, your well-being is important, and it's okay to prioritize it. Let's explore some options together and see what might work best for you.

Best regards,

Jacob

### Message-answer pair 12_4 (Human Therapist)

Patient Message

Dear Jacob

I have now had a talk with my superior about having more downtime, and he seemed fine with me taking more breaks. So I will try that.

I am, however, really stressed about going to a wedding this Sunday. I already feel that it is going to be a real shitshow. I am already wasted after my work week and just know that the next week will be even worse when I don't get a chance to recover at the weekend.

Im considering to just pull out in order to be able to focus on one thing at the time. This will however mean another fight with Mathilde who hates that we again and again have to disappoint due to my tiredness. Maybe I should just go and then try to take some breaks while we are there.

Best regards

Gustav

Therapist response

Dear Gustav,

It sounds really good that you've talked to your boss about taking more breaks and that you are going to try to use this. It's an important step towards changing the way you are at work so you don't overexert yourself.

I can see that you are ambivalent about going to the wedding. But just as you want to try to take more breaks at work, I think it would be sensible to try to use the same healthy strategy in your social life, which you also mentioned in your message. Perhaps it could be an idea to think that every hour you leave the room for 10 minutes and offer yourself some peace of mind and a different position for your body. In the same way, you could perhaps challenge how you are in the context of the party by warning in advance that you will probably leave a little early due to your functional disorder. In this way you avoid missing a festive event, while at the same time adapting what the day requires of you so that you can be there in a good way. The way you take breaks will probably need to be "hacked" to your needs and what can be done. Perhaps you could also talk to Matilde about trying something new and whether she would like to help you and support you in that?

Best

Jacob

### Message-answer pair 16_10 (LLM)

Patient Message

Dear Helene

Thank you for the talk the other day. It was nice, and I think that I have a bit more energy now and am ready to start with the program. I have talked with my parents about getting more support from home care. However, it will still be me who has to push for it and make it happen so until this is in place, I am still responsible. It has, however, given me a bit more energy to have voiced how I feel and to know that they are open for getting help from other people.

I have read a little about exposure in module 5. I am not sure what is the best thing to do. I do not feel like exercising a lot because it has previously made me feel so bad, and I just dont have the enrgy right now if I also have to get through all my day-to-day tasks. You have any ideas?

Therapist response

Dear Pernille,

It's great to hear that you're feeling a bit more energized and motivated to start working on the program! Congratulations on taking the step to talk to your parents about getting more support from home care - that takes a lot of courage. It's understandable that you still feel responsible for making it happen, but knowing that they're open to getting help from others can be a huge relief.

Regarding exposure in module 5, I completely understand your concerns. It's natural to feel hesitant about exercising or doing activities that have previously made you feel bad. The key is to start small and focus on gradual, manageable steps. We're not aiming for extreme changes or pushing yourself too hard, especially when you're still adjusting to your daily tasks.

Here's an idea: instead of focusing on exercise, what if we explore other areas where you could start with small exposure exercises? For example, are there any activities or tasks that you've been avoiding due to fear or discomfort? Perhaps we could identify one or two things that you could start with, and break them down into smaller, achievable steps.

Remember, exposure is not about pushing through pain or discomfort, but about gradually facing your fears and building confidence. We can work together to find a pace that feels comfortable for you. Let's discuss this further and see what we can come up with.

How does that sound?

### Message-answer pair 17_10 (Human Therapist)

Patient Message

Dear Helene

Thank you for the talk the other day. It was nice, and I think that I have a bit more energy now and am ready to start with the program. I have talked with my parents about getting more support from home care. However, it will still be me who has to push for it and make it happen so until this is in place, I am still responsible. It has, however, given me a bit more energy to have voiced how I feel and to know that they are open for getting help from other people.

I have read a little about exposure in module 5. I am not sure what is the best thing to do. I do not feel like exercising a lot because it has previously made me feel so bad, and I just dont have the enrgy right now if I also have to get through all my day-to-day tasks. You have any ideas?

Therapist response

Dear Pernille

Thank you for the conversation the other day. I am pleased to hear that what we discussed was relevant to you, and that you have the courage and energy to continue with the program here. It is also really good that you acted on your need to talk to your parents about getting more help from home care, so that in the longer term you can be relieved a bit in this regard

You write that you have read module 5 on exposure and that you are now a little unsure about what to do. You write that physical exercise is not what comes first for you and that you feel unsure about what would be good to do. I can understand that, because exposure can take many forms and go in many directions. First and foremost, however, it is relevant that what you choose to focus on is something you find relevant and important FOR YOU. If it is not exercise, then that is completely OK. It is YOUR treatment and it is you who decides what is most relevant and meaningful to work with right now. (When you get to module 7 on values, you will work in depth with this very thing).

However, I also want to point out that you are already practicing exposure - namely by being relieved of the daily care of your parents. It required some commitment to have the conversation with them, and it will require commitment to get the decision implemented. Maybe this is what you need to work on first? It may also be the case that there are some difficult feelings associated with having to hand over some of the responsibility for your parents to home care that you need to take care of? For example, a guilty conscience or worry.

There may also be other areas of your life (or in your relationships) where it might be good to work on exposure by scaling back and setting healthy boundaries? Remember that exposure can be both doing MORE of something - but also doing LESS.

Let me know what you think about the above. I'll log in again in 3 days, i.e. on Sunday.

Best regards Helene

## Section 3: Prompting template

*This* section *reproduces the prompting template used to generate the LLM stimulus material. The template assigns the model the role of therapist in the internet-based treatment programme and instructs it to reply to the patient message. It includes abbreviated versions of all 11 treatment modules as the clinical content basis and specifies a response length of 800 to 1200 characters. The bracketed fields were populated with the relevant patient information and patient message(s) for each stimulus before the prompt was submitted (see* Section *2):*

I am a researcher conducting an experiment where I need to generate a correspondence from an internet-based cognitive behavioral therapy treatment program between a patient and a therapist.

The patient has a functional somatic disorder characterized by multiple persistent physical symptoms which affects daily functioning.

The therapist’s role is to help the patient by replying to the message the patient has written.

I invite you to take on the role of the therapist and respond in a professional, empathic and helpful way to the patient.

The response need to be based on the treatment content of the internet based treatment programme. I have inserted an abbreviated version of the 11 modules of the treatment programme below. It needs to be between 800 and 1200 characters.

After the content I have inserted the patient message(s) you need to respond to and some basic information about the patient.

Treatment content:

”

**Modul 1**

You’re starting a 14-week online program designed to help you manage a functional disorder. The program focuses on gradual change, taken one step at a time, and offers tools and support to help reduce symptoms and improve your quality of life.

With guidance from your therapist, you'll explore 11 modules that teach new ways to understand and respond to your symptoms. Progress comes through small, regular efforts—not by rushing. You’ll also complete brief check-ins and occasional questionnaires to track how you’re doing.

Functional disorders, also called Bodily Distress Syndrome (BDS), stem from how the brain and body process stress and signals. Symptoms often persist because the nervous system stays in a heightened state—even when there’s no physical damage. They're not imagined, but the brain’s perception has become overly sensitive. Thankfully, these patterns can change.

The treatment focuses on several cores areas, including: understanding the condition, working to reduce stressors and achieve balance in the everyday life, and changing challenging symptom related anxiety and avoidance behaviour, helping the brain to relearn that the body is safe.

**Modul 2**

Many people with functional disorders go through long, frustrating medical investigations before receiving a diagnosis. The term *functional disorder* (also called Bodily Distress Syndrome, BDS) covers a wide range of conditions—like fibromyalgia, chronic fatigue, or irritable bowel syndrome—where symptoms are real but can’t be explained by typical medical tests. These different labels come from various medical specialties, but describe variations of the same issue. In Denmark, “functional disorder” is now used as an umbrella term for them all.

The condition can show up differently from person to person—pain, fatigue, dizziness, stomach problems, or sensory disturbances. If many body systems are affected, it’s called a multi-organ functional disorder. Regardless of the symptoms, the shared pattern is that the body’s stress and alarm systems are overactive, and the brain misinterprets normal bodily signals as signs of danger.

A functional disorder doesn’t appear out of nowhere. It tends to develop from a mix of long-term vulnerabilities, specific triggers (like illness or emotional stress), and factors that keep symptoms going—like overexertion, negative expectations, or avoidance behavior. Some people are born with higher sensitivity; others develop it over time due to life pressures, trauma, mental difficulties or pushing through despite early signs of distress. Often, symptoms persist because of patterns like “boom and bust”—doing too much on good days and crashing afterward.

Understanding your own condition means exploring what vulnerabilities you possess, what may have triggered the onset, and what could be maintaining your symptoms today. This reflection is called a *case formulation*, and it forms the basis for tailored treatment.

You're encouraged to see the bigger picture—not just one moment or cause, but how many aspects of your life may have played a role. This understanding is key to finding ways to reduce symptoms and regain control.

**Module 3**
Module three introduces practical steps for changing behavior patterns that may be maintaining your symptoms. A core focus is on stabilizing your daily rhythm to reduce stress on your nervous system. Many with functional disorders fall into a “boom and bust” pattern—doing too much on good days and crashing on bad ones. This cycle can gradually lower your overall functioning and emphasize symptoms.

Instead, treatment encourages you to shift toward a more balanced, predictable routine. That means stabilizing activity levels, avoiding overexertion on the better days and complete inactivity on the worse days. To support this, you’ll be introduced to the *goal ladder*—a tool that helps you break down larger goals into small, manageable steps, allowing you to slowly build up your capacity without overloading your body.

The module also highlights the importance of basic daily factors—sleep, nutrition, and movement. These are often disrupted by symptoms but can be improved in small ways.

You’ll also learn the *STOP exercise*, a short meditation technique to help you pause, check in with yourself, and choose what’s most helpful in the moment. It’s a simple but powerful way to notice whether you’re pushing too hard or holding back too much—and to adjust accordingly.

Ultimately, change happens one step at a time. With support from your therapist and consistent use of the goal ladder and STOP technique, you can begin making small adjustments that gradually improve your stability, confidence, and functioning.

**Modul 4**

The fourth module explores how we experience symptoms—not just as physical events, but as sensations shaped by how the brain processes information. A symptom isn’t just something that happens in the body—it’s also how the brain interprets bodily signals, and this process is influenced by thoughts, feelings, and past experiences. Especially with chronic or diffuse symptoms, what we expect and how we react can strongly affect how symptoms feel and how long they last.

Much of this happens automatically, like a mental "autopilot." Over time, we may develop fixed reactions to symptoms—lying down, avoiding activity, worrying—which might bring short-term relief, but risk reinforcing the problem long-term. Becoming aware of these patterns is a key part of change.

You learn to disrupt the autopilot using two tools: the STOP exercise (a brief pause to check in with your body and mind) and the "diamond" model, which helps break down a moment into thoughts, emotions, body sensations, and impulses to act. These tools increase awareness of how your brain is interpreting symptoms—and give you room to try something different.

The module also introduces the exercise ‘*behavioral analysis’*, which is a way to look at specific situations and examine how your actions affect you in the short and long term. Many well-meaning coping strategies—like avoiding social events or pushing through pain—offer immediate relief but may worsen symptoms over time. Recognizing these patterns, which we call avoidance behaviours, is a first step toward breaking them.

You’re encouraged to approach this process with curiosity, not judgment. By stepping back and reflecting on your reactions, you begin learning how to respond to symptoms in ways that help you heal rather than stay stuck.

**Modul 5**

In module 5, you learn how avoidance behaviour—while understandable and sometimes helpful in the short term—can end up maintaining and worsening symptoms over time. **Avoidance behavior**, happens when you consistently try to reduce discomfort by avoiding certain activities, places, or feelings. Over time, this strategy can reduce your freedom, and gives the symptoms control over your life. It also often increases your sensitivity to symptoms.

The best approach to challenge excessive avoidance is to use **exposure**—a method where you intentionally do things you would normally avoid. By gradually and safely confronting situations or sensations you associate with discomfort, you give your brain a chance to learn that these things may not be as dangerous or overwhelming as they seem. This helps reduce symptom-related fear and re-trains the nervous system.

Exposure is done in small, manageable steps. You start by identifying your specific avoidance behaviors—like resting to avoid pain, skipping social events, or over-preparing to avoid cognitive mistakes. Then, you plan **exposure exercises** that gently test the assumptions behind the behaviours, helping you discover what actually happens when you don’t utilize avoidance behaviour.

Importantly, exposure is not about pushing through or ignoring your limits. Instead, it’s about working within your **development zone**—where you're challenged, but not overwhelmed. You're encouraged to reflect on each exercise: What did you expect would happen? What actually happened? What did you learn?

This process can be uncomfortable, especially at first. It’s normal for symptoms to flare up temporarily, and for critical thoughts or doubts to arise. But with repetition, exposure helps reduce symptom related anxiety and avoidance, and builds a life less ruled by symptoms—and more guided by your values.

**Modul 6**

The sixth module introduces attention training, also known as mindfulness, as a way to help reconnect body and mind. Many people with functional disorders find it difficult to sense what is happening in their body clearly. Through regular practice, attention training helps you become more aware of how symptoms feel, how they fluctuate over time, and how thoughts and emotions can influence what you feel physically.

Rather than automatically avoiding discomfort, attention training invites you to stay with your experience in the present moment. This means noticing sensations without trying to change them, and observing your thoughts and feelings without judgment. Over time, this can make it easier to spot when you’re on autopilot and to gently steer your attention back toward what matters to you.

Practicing attention in this way also helps you respond to yourself with more kindness and less self-criticism. It’s not about finding calm or achieving inner peace, but about gaining insight into your experience and gradually becoming less reactive to symptoms. In that way, attention training can also function as a kind of exposure, helping you become more comfortable with the sensation of your body, which many with functional disorder usually try to avoid.

To deepen your understanding of your inner experience, the module reintroduces the Diamond—a tool to reflect on your bodily sensations, thoughts, emotions, and urges after a mindfulness exercise. This helps you observe patterns in how you respond and make more deliberate choices that support your well-being.

**Modul 7**

The seventh module explores the idea of values—what truly matters to you in life—and how they can help you navigate through difficult moments and stay on course. When symptoms dominate daily life, it’s easy to lose sight of the things that bring meaning and joy. Avoiding discomfort might offer temporary relief, but it can also move you further away from the relationships, activities, and goals that give your life direction.

Instead of being driven only by a wish to reduce symptoms, you’re encouraged to act in line with what is meaningful to you. Values are like a compass, pointing you toward the kind of person you want to be and the life you want to live. They’re not something you achieve once and for all, but something you move toward through the choices you make—especially on the difficult days.

First important step is to help discover which values you have, and which of these are the most important to you. From there, you can explore how these values might show up in your everyday life and begin to set goals that bring you closer to them. Acting on your values can provide motivation even when energy is low or symptoms are present—because you’re doing something that matters to you.

By becoming more aware of your values and allowing them to guide your actions, you start to build a life that feels more meaningful, regardless of your symptoms.

**Modul 8**

The eight module introduces the concept of self-compassion and how it can be used to calm the body’s stress response and support healing. Many people struggle to be kind to themselves—especially in the face of pain, fatigue, or setbacks—and may instead react with harsh self-criticism or unrealistic demands. But practicing self-compassion is not a sign of weakness or self-pity. It’s a way to care for yourself in moments of difficulty, the same way you might care for a friend.

Our nervous systems are equipped with both a stress response and a soothing system. For people with functional disorders, the stress system is often overactive, leading to more intense symptoms. Self-compassion is one way to activate the soothing system. This can help regulate stress, bring relief, and create the space needed for your body to rest and heal.

The module also explores how self-critical thoughts—often delivered by an "inner critic"—can increase stress and make symptoms worse. Learning to recognize and gently counter these thoughts is a key part of self-compassion. Rather than pushing yourself harder or criticizing your struggles, you’re encouraged to pause, offer kindness, and do something small and caring for yourself.

Self-compassion can look different for everyone: a warm drink, a quiet moment alone, saying no to an obligation, or doing something that brings joy or calm. Even positive activities—things you enjoy or that give a sense of meaning—can be powerful tools for well-being.

This module encourages you to make space for daily self-compassion practices and to reflect on what truly activates your soothing system. Self-care is not selfish—it’s the foundation for being well and showing up in life the way you want to.

**Modul 9**
In this module, you’ll take a step back and reflect on everything you’ve learned so far. You’ll revisit the key idea that your symptoms are linked to a sensitized nervous system—where normal signals from your body are misread as signs of danger. This misunderstanding sets off a stress response that can make your symptoms feel worse. Over time, your emotions, thoughts, and automatic habits—like avoiding things—can keep this cycle going.

You’ve worked on creating stable routines, lowering stress, and gradually facing the things you’ve been avoiding. Tools like the goal ladder, exposure exercises, and attention training have helped you do this. You’ve also learned how to take action based on your values, even when it’s uncomfortable—because it moves you toward what really matters in your life.

This module also looks at the internal obstacles that can make change harder. You might recognize automatic permissive thoughts like “I don’t have time” or rigid life rules like “I must do everything perfectly.” These patterns can pull your focus away from what’s important and stall your progress. Spotting and challenging these inner habits is a big part of continuing to move forward.

You’ll also take time to look at your goal ladder, notice the progress you’ve made, and celebrate even the small steps. Setbacks are part of the journey—they’re chances to learn, not signs of failure. The focus now is on seeing the bigger picture and finding lasting ways to shift the patterns that have kept your symptoms going.

**Modul 10**

This module focuses on how your relationships and social surroundings affect your experience with a functional disorder. Many people find it difficult to explain their symptoms to others—and sometimes, relationships can become strained or confusing as a result. But support from others are also often a key resource in your recovery. When the people close to you better understand what you’re going through, it can reduce isolation and make everyday challenges easier to handle.

You’re invited to reflect on how you currently talk about your condition and whether the way you explain it feels helpful. Some people choose to say very little, while others may share more—but it’s worth exploring what kind of communication supports you best. Family members, especially those you live with or are close to, can be a great help if they understand what you need. Being open about your condition, even with children, can also reduce worry or confusion.

The module also looks at the emotional challenges of accepting limitations. Many people experience feelings of guilt, shame, or the urge to hide their difficulties from others. You’re encouraged to reflect on both the parts of yourself you feel proud of and the parts you might try to keep hidden. Allowing all of these aspects to exist side by side is an important step toward greater self-acceptance.

To support better communication, you’ll be introduced to strategies that make conversations more constructive. One useful shift is moving from debates about what is “right” or “wrong” to conversations that focus on personal experience—where both you and the other person can feel heard. These kinds of conversations can strengthen relationships and create more understanding on both sides.

Finally, you’re encouraged to think about who in your life might act as a support person—someone who can help you stick with your goals, remind you of what you’ve learned, and be there during setbacks. Including others in your process can make the road to recovery feel less lonely—and more sustainable.

By opening up and allowing yourself to receive support, you take an important step toward building a life that’s not just about managing symptoms, but also about being connected, understood, and supported in what matters most to you.

**Modul 11**
This final module helps you prepare for the transition from structured treatment to everyday life. You’re invited to reflect on the journey you’ve been through—acknowledging the progress you’ve made, and also any lingering doubts or concerns you may still carry. It’s completely normal to feel both relief and uncertainty at this stage.

Throughout the program, you’ve gained tools, insights, and strategies to help you manage your symptoms and take steps toward what matters to you. Now, the focus shifts to continuing that work on your own. Managing a functional disorder is an ongoing process—like learning a new skill, it requires regular practice to maintain what you’ve built.

Even if symptoms return or life becomes challenging, you can return to what you’ve learned. Tools like behavioral analysis, goal-setting, exposure, and self-care are always available to help you stabilize and find your direction again. Recovery isn’t about never struggling—it’s about knowing how to support yourself when you do.

Your values continue to play an important role. When symptoms tempt you to avoid or withdraw, returning to your values can remind you why it’s worth staying engaged. Using tools like the goal ladder can help you translate your values into concrete steps in daily life.

The module also encourages you to think about who can support you moving forward. A support person—a friend, partner, colleague, or mentor—can help keep you on track, offer encouragement, and remind you of how far you’ve come when motivation dips.

You are encouraged to create a personal “first-aid kit”—a collection of strategies, tools, reminders, and reflections that you can turn to when things feel difficult. This might include exercises from the program, inspiring quotes, soothing activities, or a written note to yourself.

Recovery isn’t about being perfect—it’s about showing up again and again for what matters to you. Even the smallest steps in the right direction can create lasting change

“

Information about the patient:

Name: *[insert data]*

Age: *[insert data]*

Occupational status: *[insert data]*

Civil status: *[insert data]*

Patient message:

*[insert message]*

Llama response:

*[insert response]*

## Section 4: Items used for data collection

### Descriptive information:

1. What is your name?

2. How old are you?

3. Which institution is your primary affiliation to?

4. What is your e-mail?

5. Which country are you from?

6. What is your professional background? *(Psychologist; Medical doctor; Other)*

7. Are you currently clinically active? *(Yes; No; Not currently but I have previous clinical experience)*

8. Do you have any experience as a psychotherapist? *(Yes; No)*

9. How many years of clinical experience do you have?

10. Do you have any experience treating patients with Functional Somatic Disorder (FSD)? *(Yes; No)*

11. Do you have experience working clinically with internet-based cognitive behavioural therapy (iCBT)? *(Yes, for FSD; Yes, for other conditions (e.g. depression, anxiety etc.); Yes, for both FSD and other conditions; No)*

12. How qualified would you consider yourself to be in evaluating the quality of therapist responses in iCBT for FSD? *(Definitely not qualified; Not qualified; Somewhat qualified; Qualified; Very qualified)*

### Response quality:

**1. How would you estimate the overall quality of the response?** *(Very low; Low; Neither or; High; Very high)*

**2. How helpful would you rate the response to be for the patient?** *(Definitely not helpful; Probably not helpful; Maybe helpful; Probably helpful; Definitely helpful)*

**3. The response is empathetic** *(Strongly disagree; Disagree; Neither or; Agree; Strongly agree)*

**4. The response is professional** *(Strongly disagree; Disagree; Neither or; Agree; Strongly agree)*

**5a. The information provided by the therapist aligns with the treatment protocol** *(Strongly disagree; Disagree; Neither or; Agree; Strongly agree)*

5b. Need a refresher on the treatment approach used to treat FSD? Click the button to show the description from earlier.*( Show description; Hide description)*

**A primer on "One step at a time" - a treatment for functional somatic disorder**

The 14-week internet-based program One Step at a Time is designed to help people with functional somatic disorders (FSD) gradually understand their condition, reduce symptoms, and improve quality of life. Grounded in cognitive-behavioural therapy, the treatment takes a multi-factorial perspective on FSD, highlighting how biological, psychological, and social factors all interact in the development and maintenance of the condition.

Patients are encouraged to identify and make small, gradual changes to the factors that currently sustain their symptoms. Throughout the process, they receive support from a therapist who provides guidance and feedback via written communication.

*Etiological assumptions*

The program conceptualizes FSD as a condition where symptoms (e.g. pain, fatigue, or dizziness) arise due to the nervous system becoming overly sensitive, interpreting normal bodily signals as signs of danger. It describes how many with FSD have been through long medical investigations and received different labels - like fibromyalgia, chronic fatigue, or irritable bowel syndrome - but that these share the same underlying processes.

*Stabilization*

The program encourages patients to stabilize their everyday life, to achieve a more balanced daily activity level, neither engaging in excessive avoidance of activity due to symptoms or overexerting themselves to keep up with own and other expectations.

It introduces tools for gradual behavioural changes such as the goal ladder, which breaks large goals into small, manageable steps. Attention is also given to basic factors such as sleep, nutrition, and physical activity, supported by short practices like the STOP-exercise to pause and adjust activity levels in the moment.

*Exposure*

As participants gain stability, the treatment focuses on how symptoms are shaped not only by the body but also by the brain's interpretations, influenced by past experiences and automatic reactions. Exposure exercises are introduced as a tool to allowing the nervous system to relearn that ordinary sensations, activities, and situations are not as threatening as they may feel, so as to reduce symptom-related anxiety and achieve more freedom in everyday life.

*Optional modules*

A set of optional modules builds further on this foundation, as participants 1) learn mindfulness-based attention training to connect more calmly with their body, 2) explore how personal values can guide meaningful choices, 3) practice self-compassion to soothe an overactive stress response, 4) strengthen supportive relationships and 5) address fundamental assumptions that may be driving unhelpful behaviours, emotions or cognitions.

**6. Who would you guess wrote the response?** *(Human therapist; AI therapist)*

**7. Here you can write down thoughts and reflections in relation to the correspondence** (Free-text response)

## Section 5: Results of subgroup analyses

|  |  |
| --- | --- |
|  |  |
|  |  |
|  | |

*This* section *presents the subgroup analyses (a-g), with one forest plot per item. In panels a-e, the plot shows the mean difference in ratings between human and LLM-generated responses across rater subgroup variables (vertical axis), with points marking the mean difference and lines the confidence intervals. Values left of zero favour human responses, values right favour LLM responses. Panels f and g show the correct classification rate across the same rater subgroup variables.*

## Section 6: Sensitivity Analysis

|  | LLAMA | Human |  |  |  |
| --- | --- | --- | --- | --- | --- |
|  | *M (SD)* | *M (SD)* | Δ | *d* | *P* |
| 1: Quality (main) | 4.00 (0.54) | 3.96 (0.53) | 0.04 | 0.06 | .66 |
| 1: Quality (sensitivity) | 3.99 (0.54) | 3.98 (0.54) | 0.01 | 0.01 | .89 |
|  |  |  |  |  |  |
| 2: Helpfulness (main) | 3.85 (0.57) | 3.93 (0.49) | -0.08 | 0.13 | .32 |
| 2: Helpfulness (sensitivity) | 3.86 (0.59) | 3.98 (0.47) | –0.12 | 0.21 | .13 |
|  |  |  |  |  |  |
| 3: Empathy (main) | 4.31 (0.47) | 4.08 (0.50) | 0.22 | 0.42 | <.001 |
| 3: Empathy (sensitivity) | 4.28 (0.47) | 4.10 (0.50) | 0.18 | 0.35 | .01 |
|  |  |  |  |  |  |
| 4: Professionalism (main) | 4.25 (0.53) | 4.11 (0.53) | 0.14 | 0.24 | .08 |
| 4: Professionalism (sensitivity) | 4.23 (0.54) | 4.12 (0.54) | 0.1 | 0.18 | .19 |
|  |  |  |  |  |  |
| 5: Protocol adherence (main) | 4.13 (0.52) | 4.13 (0.54) | 0.01 | 0.03 | .81 |
| 5: Protocol adherence (sensitivity) | 4.16 (0.54) | 4.17 (0.53) | –0.01 | 0.02 | .85 |
|  |  |  |  |  |  |
| 6: Correct classifciation (%) (main) | 62.72 (21.30) | 78.64 (19.65) | –15.92 | 0.79 | <.001 |
| 6: Correct classifciation (%) (sensitivity) | 63.00 (21.98) | 78.51 (20.23) | –15.51 | 0.78 | <.001 |

**Abbreviations:** M, mean; SD, standard deviation; Δ, mean difference between LLM and Human; P, significance level of t-test. Note: Sensitivity analyses exclude all individuals (n = 5) who scored themselves as not currently clinically active.

## Section 7: Characteristics of responders to open-ended text fields

| **Characteristics** | *n(%)* |
| --- | --- |
| **Country of residence** |  |
| Belgium | 1 (3%) |
| Denmark | 16 (53%) |
| Germany | 2 (6%) |
| Sweden | 11 (36%) |
| **Professional background** |  |
| Psychologist | 24 (80%) |
| Medical doctor | 6 (20%) |
| **Currently clinically active** |  |
| Yes | 28 (93%) |
| No | 2 (6%) |
| **Experience as psychotherapist** |  |
| Yes | 28 (93%) |
| No | 2 (6%) |
| **FSD treatment experience** |  |
| Yes | 23 (76%) |
| No | 7 (23%) |
| **iCBT experience** |  |
| ICBT experience with FSD | 9 (30%) |
| ICBT experience with other conditions | 9 (30%) |
| No ICBT experience | 12 (40%) |
| **Self-rated qualification to evaluate psychotherapy exchanges** | |
| Definitely not qualified | 1 (3%) |
| Somewhat qualified | 8 (26%) |
| Qualified | 15 (50%) |
| Very qualified | 6 (20%) |
|  | *Mean (SD)* |
| **Age (years)** | 39.83 (8.98) |
| **Years of clinical experience (years)** | 11 (8.5) |

**Note**: Descriptive characteristics of raters who provided comments in one or more open-ended text fields.

## Section 8: Ratings on the main outcome variables of responders to the qualitative analysis

|  | LLAMA | Human |  |  |
| --- | --- | --- | --- | --- |
|  | *M (SD)* | *M (SD)* | Δ | *P* |
| **1: Quality** | 3.91 (0.56) | 3.96 (0.60) | –0.05 | .67 |
| **2: Helpfulness** | 3.85 (0.66) | 4.06 (0.50) | –0.21 | .06 |
| **3: Empathy** | 4.26 (0.53) | 4.13 (0.52) | 0.13 | .16 |
| **4: Professionalism** | 4.14 (0.56) | 4.07 (0.53) | 0.06 | .55 |
| **5: Protocol adherence** | 4.03 (0.55) | 4.16 (0.50) | –0.13 | .14 |
| **6: Correct classification (%)** | 64.23 (18.49) | 80.23 (20.14) | –16.00 | <.001 |

**Abbreviations:** M, mean; SD, standard deviation; Δ, mean difference between LLM and Human; P, significance level of t-test.

## Section 9: Codebook from qualitative analysis

| **Codes** |  |  |  |
| --- | --- | --- | --- |
| Level 1 | Level 2 | Level 3 | Times Used |
| AI CODES |  |  |  |
|  | AI: misc |  | 0 |
|  |  | AI: AI/HM value statement | 5 |
|  |  | AI: CBT vs. iCBT | 1 |
|  |  | AI: Cultural fit | 2 |
|  |  | AI: professional opinion | 2 |
|  | AI: negative |  | 21 |
|  |  | AI: inauthentic, AI-ish | 10 |
|  |  | AI: lack of structure | 3 |
|  |  | AI: not challenging enough | 22 |
|  |  | AI: not empathetic enough | 4 |
|  |  | AI: not professional | 2 |
|  |  | AI: too advisory | 2 |
|  |  | AI: too assuming | 4 |
|  |  | AI: too empathetic/supportive | 15 |
|  |  | AI: too little protocol adherence | 6 |
|  |  | AI: too long response | 10 |
|  |  | AI: too many questions/overwhelming | 5 |
|  |  | AI: too shallow/unconcrete/generic | 32 |
|  |  | AI: unhelpful/problematic advice | 17 |
|  | AI: positive |  | 19 |
|  |  | AI: empathetic | 9 |
|  |  | AI: good quality | 19 |
|  |  | AI: good/helpful/concrete advice | 8 |
|  |  | AI: invites reflection | 3 |
|  |  | AI: personal response | 2 |
|  |  | AI: positively challenging | 3 |
|  |  | AI: protocol allignment | 3 |
|  |  | AI: well- written/structured | 4 |
|  | AI: source |  | 0 |
|  |  | AI: AI suspicion | 16 |
|  |  | AI: human suspicion | 4 |
| HUMAN CODES |  |  |  |
|  | HM: AI HM value statement |  | 17 |
|  | HM: CBT vs. iCBT |  | 1 |
|  | HM: Negative |  | 18 |
|  |  | HM: Too professional | 1 |
|  |  | HM: incorrect thera. comp | 1 |
|  |  | HM: low protocol adherence | 6 |
|  |  | HM: message unclear | 6 |
|  |  | HM: not challenging enough | 1 |
|  |  | HM: problems with style/structure | 15 |
|  |  | HM: too advicing/directive | 5 |
|  |  | HM: too assuming | 1 |
|  |  | HM: too eager to intervene | 3 |
|  |  | HM: too friendly/empathetic | 8 |
|  |  | HM: too informal/unprofessional | 9 |
|  |  | HM: too little empathy | 7 |
|  |  | HM: too long | 11 |
|  |  | HM: too shallow/unspecific | 10 |
|  |  | HM: unhelpful/problematic | 4 |
|  | HM: Positive |  | 32 |
|  |  | HM: Personalized | 10 |
|  |  | HM: authentic | 2 |
|  |  | HM: challenging | 5 |
|  |  | HM: message well structured | 8 |
|  |  | HM: message/advice concrete | 14 |
|  |  | HM: protocol/component adherence | 9 |
|  |  | HM: provides insight | 5 |
|  |  | HM: seems useful | 6 |
|  |  | HM: short message | 3 |
|  |  | HM: supportive/empathetic | 18 |
|  |  | HM: misc. | 0 |
|  | HM: salient feature of message |  | 2 |
|  | HM: source |  | 0 |
|  |  | HM: AI suspicion | 2 |
|  |  | HM: human suspicion | 36 |

**Note:** This section presents the codebook from the qualitative analysis of raters' free-text comments. Codes are organised across three nested levels, from the broadest category (Level 1) to the most specific (Level 3): Level 1 distinguishes comments on AI-generated from human therapist-generated (HM) responses; Level 2 groups these into broader categories (e.g. positive, negative, source); and Level 3 holds the specific codes. 'Times Used' reports how often each code was applied, with Level 1 and Level 2 counts reflecting codes applied directly at that level rather than the sum of their subordinate codes.

**Abbreviations:** HM, human; AI, Artificial intelligence.
